# PET/MRI in infectious and inflammatory diseases: will it be a useful improvement?

**DOI:** 10.1007/s00259-012-2060-9

**Published:** 2012-02-02

**Authors:** Andor W. J. M. Glaudemans, Ana Maria Quintero, Alberto Signore

**Affiliations:** 1 Department of Nuclear Medicine and Molecular Imaging, University Medical Center Groningen, University of Groningen, Groningen, the Netherlands; 2 Department of Radiology, Clinica Colsanitas S.A., Bogotà, Colombia; 3 Nuclear Medicine Unit, Faculty of Medicine and Psychology, “Sapienza” University of Rome, Rome, Italy; 4 Medicina Nucleare, Ospedale S. Andrea, University of Rome “Sapienza”, Via di Grottarossa 1035, 00189 Roma, Italy

The knowledge of disease mechanisms and the identification of markers of disease development and progression, and for therapy evaluation, are rapidly expanding. Early diagnosis, possible disease prevention and individual therapy stratification are important goals in health care. Nuclear medicine and radiology are potentially able to satisfy these increasing demands for diagnosis, prevention, pathophysiological understanding and treatment possibilities. An increasing number of biomarkers, drugs, antibodies and peptides, contrast agents and MRI sequences have also been developed for these purposes. Together with this continuing search for the “holy grail” radiopharmaceutical, the use of hybrid imaging is another important development in the field. A recent development in hybrid cameras is the introduction of combined PET and MRI. Despite the intriguing novelty, it is important to evaluate carefully, in a cost–benefit manner, the possible advantages and clinical applications of such an expensive tool.

## Advantages in general of hybrid PET/MRI systems

While PET enables the acquisition of functional data at the molecular level, MRI provides superior soft-tissue resolution and anatomy along with semiquantitative macromolecular information. Hybrid PET/MRI systems can combine these imaging characteristics. PET/MRI systems can be either simultaneous or sequential. Although imaging data from separate PET and MRI systems may be combined, simultaneous imaging systems have major theoretical advantages that could be of interest to the whole medical community. The general advantages of a simultaneous PET/MRI system as compared to conventional PET/CT systems are:

- Recording of dynamic and moving phenomena, which could enable tracers with short half-lives to be studied
- Identical position of the patient during image acquisition with both modalities leading to a substantial reduction in motion artefacts due to heart beating, intestinal motion and breathing.
- Absolute match between the tissue information of both modalities under the same physiological conditions.
- Contrast-enhanced MRI information (e.g. on perfusion and blood flow) can be used in the pharmacokinetic modelling of the PET data, resulting in an improvement in PET reconstruction and data analysis.
- Better localization of the PET signal within the soft tissues.
- No radiation burden from the MRI part.
- Better application of the one-stop-shop principle for simultaneous diagnostic-quality acquisition of nuclear medicine and radiological images.
- More structural, functional and molecular imaging at the same time.

On the other hand, compared to PET/CT, PET/MRI examinations may result in a lower compliance by the patient, because of long examination times and noise. Furthermore, MRI cannot image all body parts at once (different body parts may require different MR acquisition sequences and probes). This new hybrid technique may therefore require collaboration between radiologists and nuclear medicine physicians for image interpretation and might be more expensive than PET/CT scans (both capital and running costs). It is therefore of economical and clinical importance to correctly identify the possible fields of relevance of PET/MRI over other imaging techniques, especially PET/CT.

Our aim here was not to provide a cost–benefit analysis of PET/MRI, but to highlight some possible fields of application in which PET/MRI could be of great clinical relevance.

## PET/MRI in infectious and inflammatory diseases

Until recently, functional imaging of inflammation and infection was mainly restricted to planar imaging and SPECT. More and more PET radiopharmaceuticals have now become available allowing more sensitive detection and quantification of specific aspects of inflammatory processes. There is substantial potential in the application of hybrid whole-body PET/MRI to the investigation of infectious and inflammatory diseases.

PET can give functional information about the target organ, including disease activity, release of cytokines and infiltration of specific immune cell populations. However, PET does not provide anatomical information, which can seriously hamper accurate diagnosis. The use of hybrid PET/CT is of limited added value in most cases of inflammation, since it lacks good soft-tissue contrast. MRI offers better anatomical and tissue contrast information, and macromolecular functional information. Simultaneous acquisition of PET and MRI could overcome these shortcomings by providing quantitative molecular functional information concerning the inflammatory lesion, and accurate localization as well as anatomical changes with motion correction. This could improve differential diagnosis and guide antiinflammatory treatment strategies, resulting in better medical care.

The combination of PET radiopharmaceuticals and PET/MRI imaging could significantly improve the sensitivity and specificity of the diagnosis and follow-up treatment of infectious and inflammatory diseases. It would allow more accurate assessment of the extent and exact localization of inflammatory lesions than PET alone or PET/CT, especially in soft tissues that are prone to movement artefacts, for example in vascular and cardiac infections, inflammatory bowel disease (IBD) and diabetes. MRI now offers more than just anatomical information, with functional MRI (fMRI), including diffusion-weighted imaging (DWI), magnetic resonance spectroscopy (MRS) and perfusion imaging. There have also been improvements in MRI contrast agents which can be used with radiolabelled probes that may lead to even more insights into the dynamics and characteristics of the inflammatory process. A third interesting feature of PET/MRI is motion correction based on MRI, which would allow more accurate quantification of PET data, leading to better treatment monitoring and the possibility of earlier response evaluation.

We now describe some relevant infectious and inflammatory diseases in which we foresee a clinical impact of PET/MRI.

### Type 1 diabetes mellitus

Insulin-dependent diabetes mellitus (IDDM) results from the cell-mediated autoimmune destruction of beta cells of the pancreas. The autoimmune process, called insulitis, precedes the clinical onset of type 1 diabetes. When clinical symptoms are observed, the autoimmune process is markedly advanced: 60–80% of the pancreas has been destroyed at the time of diagnosis [1]. Several attempts to image insulitis and the beta-cell mass have been made with both CT and MRI [2]. The MRI approaches to the study of IDDM have mainly been developed on animal models; a few studies have used magnetic nanoparticles as a contrast agent for MRI [3]. Similarly, several PET and SPECT tracers, such as ^18^F-FDG, labelled leucocytes, antibodies and immunoglobulins have been used to try to detect insulitis [4–9]. However, none of these tracers and imaging modalities are able to target specifically the inflammatory process involved in insulitis.

Interleukin-2 (IL-2) labelled with ^99m^Tc or ^123^I is a SPECT radiopharmaceutical specific for the IL-2 receptor that is overexpressed in infiltrating lymphocytes during the prediabetic phase. Both tracers have been studied in animal models of insulitis and in patients with newly diagnosed type 1 diabetes [9–12]. The results were promising, but the signal in the pancreas was still too low to be able to obtain quantitative data. Therefore, the new PET tracer *N*-(4-[^18^F]fluorobenzyl)interleukin-2 (^18^F-IL-2) was developed, a tracer that is able to target specifically CD25-positive cells. ^18^F-IL-2 is a promising tracer for insulitis imaging because it combines the specific targeting of activated T-lymphocytes and the high spatial resolution provided by PET. This tracer has already been tested in diabetic prone rats and mice with good results in terms of localization and quantification of insulitis.

MRI is now the noninvasive imaging tool of choice with high spatial resolution and the highest soft-tissue contrast; however it does not give any functional information. In contrast, PET can give information about the physiological status of the particular target organ. A disadvantage of PET is that peristaltic motion of the abdominal organs, including the pancreas, can cause artefacts in the PET image. PET/MRI may be able to solve this problem by using the MRI data to correct the artefacts in the PET imaging. In the case of insulitis, combined and simultaneous PET/MRI imaging could provide the exact anatomical localization and the inflammatory status of the pancreas, and this technique may provide a unique opportunity to quantify insulitis in humans. Thus, following further development, this method could improve the diagnostic accuracy of insulitis imaging and could be used in the routine follow-up of diabetic patients.

To summarize, PET/MRI in IDDM may provide the opportunity to:

- Visualize the cell-mediated autoimmune destruction of beta cells of the pancreas (insulitis) at an early stage
- Quantify insulitis at an early stage
- Accurately diagnose insulitis and routinely follow-up diabetic patients.

### Spondylodiscitis

Spondylodiscitis is the main manifestation of haematogenous osteomyelitis and represents 3–5% of all cases of osteomyelitis [13]. The incidence has risen over the years as a result of an increase in the susceptible population (increase in average age, immunodeficiency, chronic steroid use, infections associated with health care), an increase in the number of patients undergoing spinal surgery, and better diagnostic possibilities. Diagnosis is often delayed due to the insidious nature of symptom onset and the high frequency of low back pain in the general population [14]. The literature refers to an average diagnostic delay of 2–6 months from the onset of clinical signs [13]. Diagnosis of spondylodiscitis is based on clinical, laboratory and radiological features and is often difficult to establish.

MRI is the modality of choice when clinicians have a suspicion of a spinal infection. MRI has advantages over CT because it can better distinguish bone marrow, vertebra and intervertebral disc, is better able to evaluate the neural structures (including the spinal cord, epidural space and peripheral soft tissue) and uses no radiation. Good sensitivity and specificity data have been reported for the use of MRI in spondylodiscitis [15]. Therefore, MRI can currently be considered the best conventional imaging method for diagnosis. However, the results for the use of MRI for treatment response monitoring in patients with spinal infections are less clear.

In nuclear medicine, ^18^F-FDG is nowadays considered the gold standard technique to detect spondylodiscitis and to evaluate therapy success since radiolabelled white blood cells do not migrate in the infected intervertebral discs and vertebral bodies due to modification of the portal-like vascularization as a result of oedema associated with the infection. The limitation of FDG its the non-ability to differentiate between spondylodiscitis and tumour (metastases) and the limited spatial resolution (not possible to distinguish between vertebra, intervertebral disc and bone marrow).

The combination and strengths of both modalities combined in a simultaneous PET/MRI study could be of relevance in patients with suspected spondylodiscitis. The main goals are:

- To assess if sensitivity and specificity of the diagnosis will improve by combining PET and MRI simultaneously
- To assess if this hybrid imaging could be used for follow-up during treatment

### Inflammatory bowel diseases

IBD are represented mainly by two disorders: ulcerative colitis and Crohn’s disease. A key role in the development of these diseases is played by the infiltration and activation of leucocytes, macrophages and T-cells [16]. These cells and other molecular markers in the process such as chemokines, cytokines and receptors of the immune response system can be used as markers for scintigraphic imaging. At the moment, colonoscopy and/or small-bowel follow through are considered by most to be the gold standard imaging techniques for the diagnosis of IBD. However, since the majority of patients need long-term follow-up it would be ideal to rely on a noninvasive technique with good compliance.

With the possibility of intravascular specific contrast agents for blood enhancement, CT, ultrasonography and MRI are all able to detect increases in biological or endoscopic signs of disease activity (vessel dilation, wall thickening, wall stratification with thickening of the submucosa, wall and mesenteric hypervascularity, lymph-node enlargement and enhancement). In comparison with CT and ultrasonography, MRI has shown a higher sensitivity in detecting these signs of intestinal and mesenteric inflammation. MRI has an intrinsically higher soft-tissue contrast and sensitivity for inflammatory abdominal tissues [17]; moreover, signs of inflammation can be detected and analysed using different MRI parameters. T1-weighted MRI parameters are similar to those in contrast-enhanced CT or contrast-enhanced ultrasonography, whereas other parameters are absolutely specific for MRI, such as T2-weighted or diffusion imaging parameters. In general, both T1- and T2-weighted MRI parameters have been successfully and reliably correlated with signs of active Crohn’s disease [18, 19]. The potential of the newest developments in MRI techniques, such as MRS, DWI and molecular imaging, is still unknown. MRS is used to measure the levels of different metabolites in body tissues. The characteristics of the resulting spectra can be used to diagnose metabolic disorders [20].

DWI expresses molecular diffusion, which is the thermally induced Brownian motion of water molecules, without the administration of any contrast agent [18]. DWI is used in solid organs such as the liver, pancreas, spleen and kidney. DWI has not been commonly used for imaging of the intestinal tract because image quality is severely degraded by motion artefacts related to intestinal motion. However, recently free-breathing DWI sequences which are associated with fewer motion artefacts have been introduced. This technique may allow the evaluation of IBD. DWI has shown the ability to differentiate active from inactive disease, as well to perform a quantitative analysis of disease activity [21].

Huge advantages from the use of hybrid PET/MRI systems in IBD may be expected. Cells can be labelled with radionuclides, with fluorescent or bioluminescent markers (optical imaging) or with MRI contrast agents, defined as molecular MRI [22]. The combined use of nuclear medicine techniques (PET for imaging cells and molecular events involved in the disease) and MRI (for morphological definition of affected bowel segments) is the key for the future approach to IBD. We foresee further developments in molecular MRI combined with nuclear medicine imaging, which could be effectively used in the detection of active inflammatory cells and cytokines in IBD.

The main goal for PET/MRI in IBD therefore should be to assess if combined PET/MRI using targeted molecular imaging and different MRI techniques (MRS, DWI) is able to detect IBD with high accuracy.

### Diabetic foot infection

Foot infections are one of the most common and severe complications of diabetes mellitus. Detection, however, can be difficult. On clinical examination it is difficult to differentiate between soft-tissue infection and osteomyelitis. Infection parameters in the blood (erythrocyte sedimentation rate, C-reactive protein) are not specific. Bone biopsy is not always performed because it is an invasive procedure that loses its reliability if the biopsy fragments are contaminated by cutaneous bacteria. Therefore, imaging is crucial in diagnosing and evaluating diabetic foot infections. Plain radiography and CT are routinely used, but are not accurate enough. MRI is able to differentiate between osteomyelitis and soft-tissue infection, but the specificity is reduced if bony destruction, dislocation, marrow oedema, synovial effusion and loss of bone and joint limits are present (which are characteristic of neuropathic Charcot’s joints) as well as osteomyelitis.

Nuclear medicine techniques play an important role in the diagnosis of diabetic foot infections. White blood cell scintigraphy with either ^99m^Tc-exametazime (HMPAO) or ^111^In-oxine is currently the gold standard radionuclide technique for the diagnosis of osteomyelitis [23]. The labelling method is well described in several guidelines [24, 25]. Another possibility is the use of ^18^F-FDG, but routine FDG PET protocols cannot reliably distinguish infection from inflammation. No acquisition protocols for FDG PET in the diabetic foot have yet been validated and only a few studies comparing FDG PET and white blood cell scintigraphy are available [26].

Combining MRI and nuclear medicine imaging may help improve the accuracy in diagnosing diabetic foot infections. The sensitivity of MRI alone or FDG PET alone is not high enough, but as for PET/CT, we think combined imaging improves sensitivity as well as specificity.

Therefore, in comparison with PET or MRI alone, hybrid PET/MRI imaging could:

- Improve accuracy in the diagnosis of diabetic foot infection
- Improve differentiation of osteomyelitis, Charcot’s joint and soft-tissue infections

## Other possibilities for the use of MRI in infectious and inflammatory diseases

Over the past decade, MRI has gained importance in the evaluation of infectious and inflammatory diseases. On T2-weighted images, infiltrated tissues exhibit a hyperintense signal which resolves after successful treatment. Thus, MRI allows follow-up of lesion development in time and space. However, as an important constraint, MRI only reflects nonspecific proton changes caused by a variety of different processes rather than just tissue infiltration by mononuclear cells. It does not visualize histological details such as the accumulation of inflammatory cells in nuclear medicine. Therefore, there is a demand for further developments in the field of molecular MRI with the use of new contrast agents and fMRI sequences.

## Conclusion

The ability to image inflammatory processes at the molecular and cellular levels would dramatically improve diagnostic capabilities as well as provide in-depth data for new drug development and biological understanding of the inflammatory processes under different conditions. MRI contrast agents to enhance the detection of inflammation and fMRI are under development. For PET, several radiopharmaceuticals are already in clinical use with high sensitivity and specificity but with relatively low spatial resolution, although some studies have found the technique sufficient for inflammation localization. However, this approach has not been widely embraced for monitoring therapeutic responses.

We have only mentioned some inflammatory indications in which hybrid PET/MRI imaging could help solve diagnostic and evaluation problems. There are of course many more diseases in which PET/MRI could be helpful, e.g. autoimmune pancreatitis, rheumatoid arthritis, transplant rejection, vasculitides, inflammatory brain disorders, and pericarditis.

This editorial emphasizes the emerging importance of PET/MRI in the field of inflammation and infection imaging, but we also emphasize the intrinsic difficulties in the use this approach in humans. The future is to combine PET imaging with MRI not only to increase anatomical resolution but also for cell and molecular imaging, and to bring the technology to the highest possible stage of development so that the potential of these two unique modalities in combination can be realized.

We leave others to calculate the cost–benefit ratio of PET/MRI in these diseases, but from the medical and scientific point of view the challenge is high.
